# Performance of the counter-torque technique in the explantation of nonmobile dental implants

**DOI:** 10.1186/s40729-019-0197-z

**Published:** 2020-01-09

**Authors:** Eduardo Anitua, Sofia Fernandez-de-Retana, Mohammad H. Alkhraisat

**Affiliations:** 1 Clínica Eduardo Anitua, Vitoria, Spain; 2 BTI Biotechnology Institute, Vitoria, Spain; 3 Eduardo Anitua Foundation, C/Jose Maria Cagigal 19, 01007 Vitoria, Spain

**Keywords:** Explantation, Counter/torque, Dental implant, Periimplantitis, Implant removal, Minimally invasive

## Abstract

**Background:**

The application of the counter-torque technique has been proposed as a conservative and atraumatic alternative for the explantation of nonmobile dental implants. The objective of this report is to assess the performance of this technique in a large number of patients.

**Results:**

Three hundred and fifty-five patients were treated for the explantation of 749 nonmobile dental implants. The explantations were performed by the application of counter-torque to break the bone-implant interface. Successful implant explantation was achieved in 98.4% of the implants. The frequency of complications was 1.3%, most commonly related to the appearance of fissure lines at the implant neck.

**Conclusions:**

The counter-torque technique has a high success rate but is not exempt from complications, although at a very low rate.

## Background

Although presenting high and acceptable survival rates [1, 2], dental implants fail as a consequence of infection (periimplantitis), excessive biomechanical stress, or improper positioning. Different techniques have been described to remove a failed dental implant that include block resection, buccal bone osteotomy, trephine osteotomy, and piezosurgery [3, 4]. The application of counter-torque to break the implant-bone interface has been proposed as a safe, efficient, and atraumatic strategy to remove nonmobile implants [4]. As a minimally invasive technique, Solderer et al. have recommended the counter-torque technique as the first option for the removal of failed nonmobile dental implants [5]. Nevertheless, there is a need of studies with a larger sample size to confirm the efficacy and safety profile of this strategy. This study aims to assess the performance of the counter-torque strategy for the removal of a large number of nonmobile dental implants.

## Methods

Patients of legal age treated for implant explantation of nonmobile dental implants between March 2010 and December 2018 were included in this retrospective study.

The treatment was performed using an implant removal kit (BTI Biotechnology Institute, Vitoria, Spain) that allowed the application of a counter-torque to the bone-implant interface [4]. The decision to raise a flap or not was made according to the surgical needs. Following the manufacturer instructions, a ratchet was engaged into the implant connection and then a counter-torque was exerted by a wrench in counter-clockwise direction. If the counter-torque exceeded the 200 Ncm limit (the torque wrench opened), trephine bur was used to cut into the first 3–4 mm of implant-bone contact. The implant explantation was then continued with the torque wrench. Treatment was considered a failure if the implant could not be removed by this procedure. All the complications occurring during the procedure were also recorded.

The cause of implant removal was grouped into biological (excessive bone loss), mechanical (improper implant positioning, prosthetic failure), and surgical (treatment of lesion that involve dental implant like medication-related osteonecrosis of the jaw).

The bone level around the implant before the explantation was measured on panoramic radiographs (Sidexis XG, Sirona Dental Systems). The percentage of the implant length covered by bone was measured mesially and distally to the implant by considering the implant length as the 100%. Then the mean of the two measurements was calculated. The frequency of the implant covered by bone was grouped into four different categories 0–25%, 26–50%, 51–75%, and 76–100%. It was not possible to calculate the implant length covered by bone in millimeters as the implant length was unknown for most of the implants.

### Statistical analysis

The frequency of the variables of interest (implant location, cause of implant removal, implant length covered by bone, implant removal success, and complications) was calculated and represented in percentage. Statistical analysis of categorical variables was calculated using the Chi-square test. The IBM SPSS Statistic v15 software package (SPSS Inc., Chicago, IL, USA) was used. Statistical significance was set at *p* value < 0.05.

## Results

**Fig. 1:**
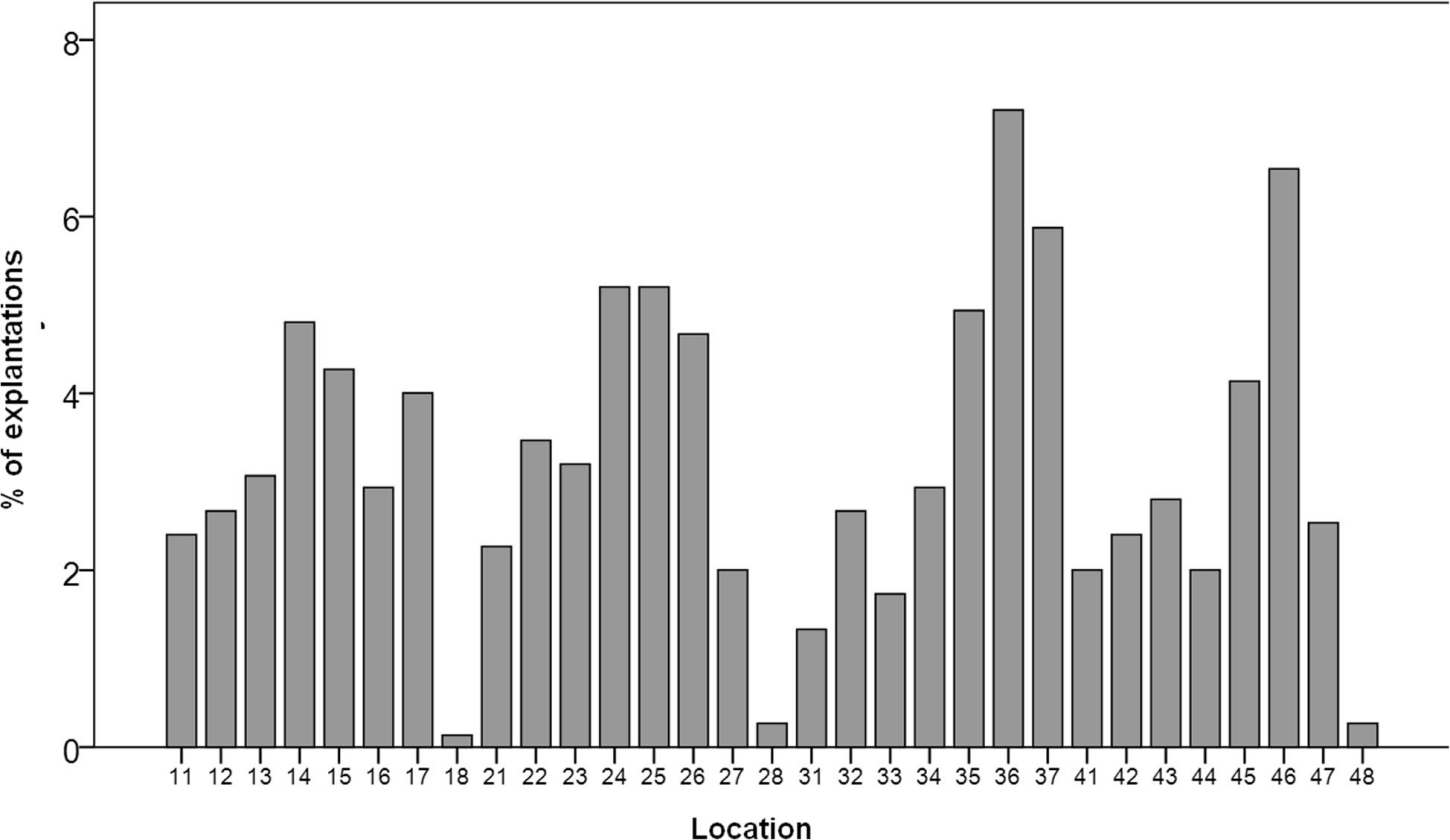
Frequency distribution of the location of the explanted dental implant

**Fig. 2:**
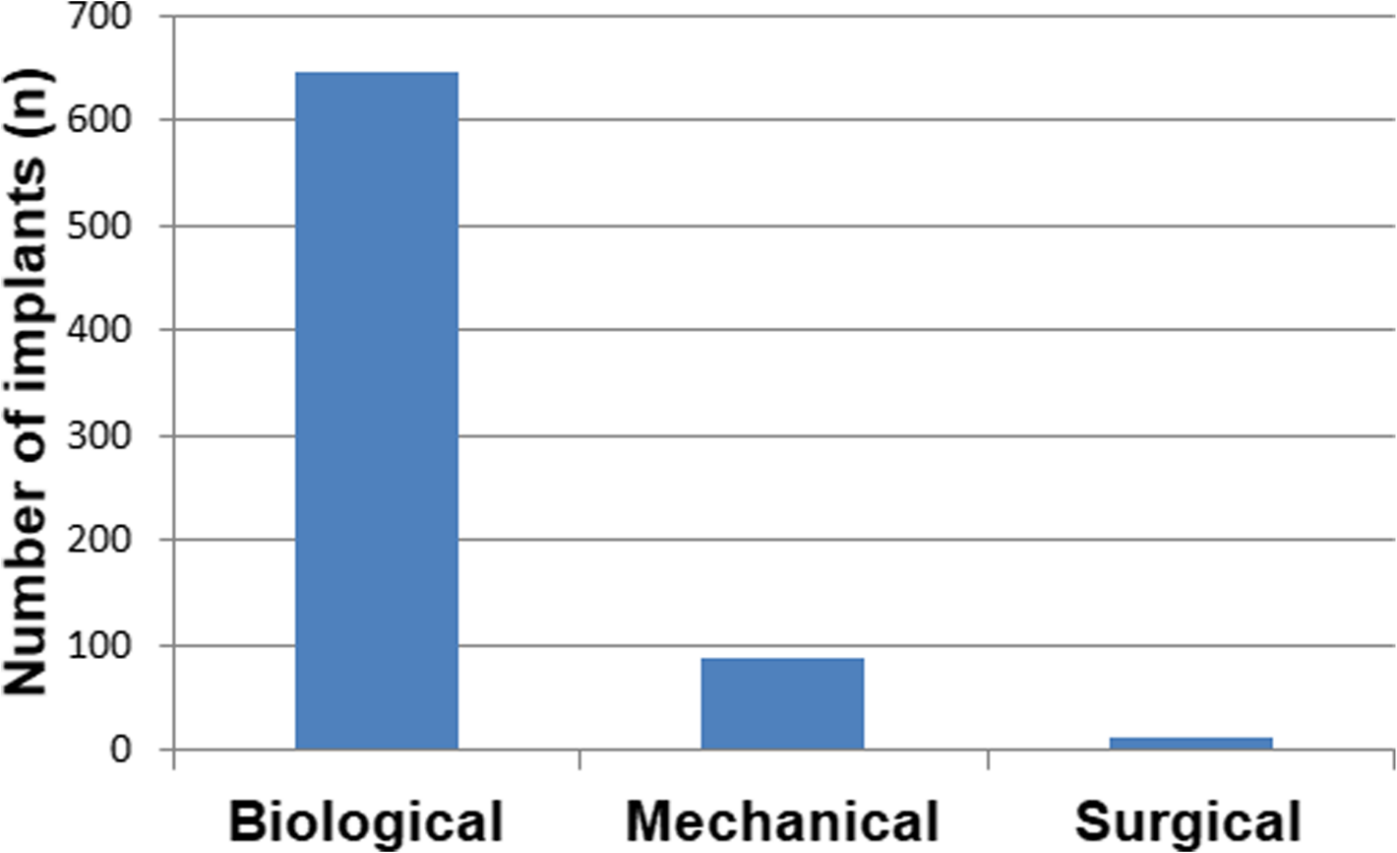
The cause of implant removal

In this report, 749 nonmobile dental implants were explanted in 355 patients. Figure 1 shows the anatomical location of the dental implants. The 50.6% of the removed implants were located in the maxilla and 48.2% of the cases were located in the anterior areas. Attending to the causes of explantation, the vast majority of the explantations occurred due to biological complications (86.2%), followed by mechanical complications (11.9%) and surgical intervention (1.9%). Figure 2 shows the distribution of the dental implants according to the cause of implant removal.

The bone level of the explanted implants was homogenously distributed among bone level categories. In this sense, 26.4% of the implants were included in the 0–25% of bone level category, 24.3% in the 26–50% of bone level category, 26.9% in the 51–75% of bone level category and, finally, 22.4% of the explanted implants were in the 76–100% bone level category. The implants were completely covered by bone (100%) in the 76–100% of bone level category were removed due to mechanical problems.

Regarding the success rate, 98.4% of the dental implants were successfully removed following the described procedure. Among these successful explantations, the use of specialized trephine burs was needed in 7.5% of the cases. It was studied if the bone level was associated with the use of trephine burs. There was no statistically significant (*p* = 0.249) relationship between trephine bar use and bone level.

The extraction kit also demonstrated to cause minimal complications that occurred in 1.3% of the extracted implants. All the detected complications were identified as fractures of the implant. There was no detection of other side effects. From the detected implant fractures, 50% were identified as fissure lines at the implant neck but allowed the successful removal of the implant. The other implant fractures were located at the apical third of the implant body.

**Fig. 3:**
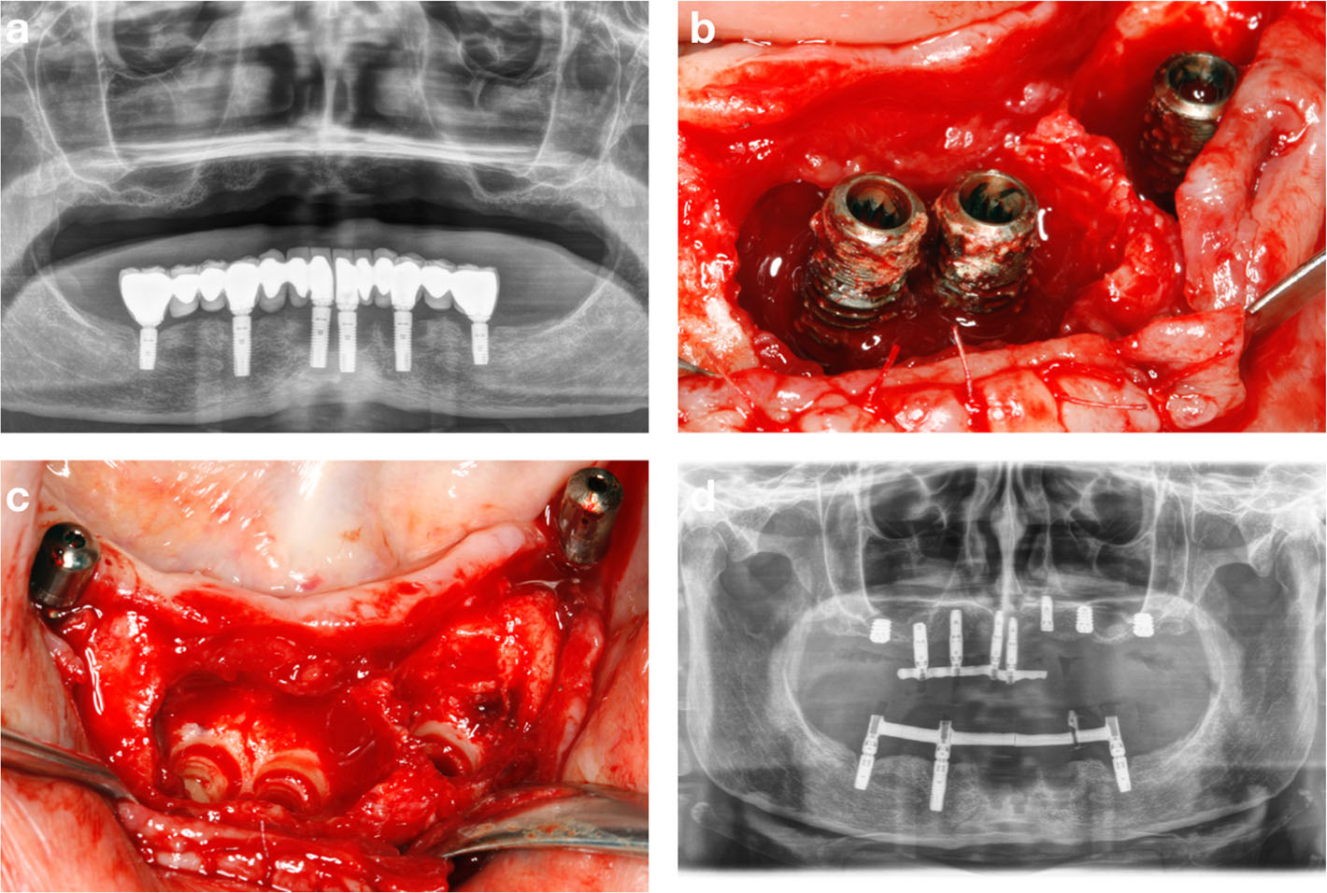
Panoramic radiograph showing excessive marginal bone loss affecting all the dental implants in the mandible supporting fixed prostheses (**a**). Clinical image showing the advanced bone destruction around the implants at the incisors and left premolar regions (**b**). Clinical image showing the preservation of the pre-existing bone upon implant removal with the counter-torque regions (**c**). Panoramic radiograph showing the maintenance at this stage of 3 implants to support the provisional prosthesis in the mandible (**d**)

**Fig. 4:**
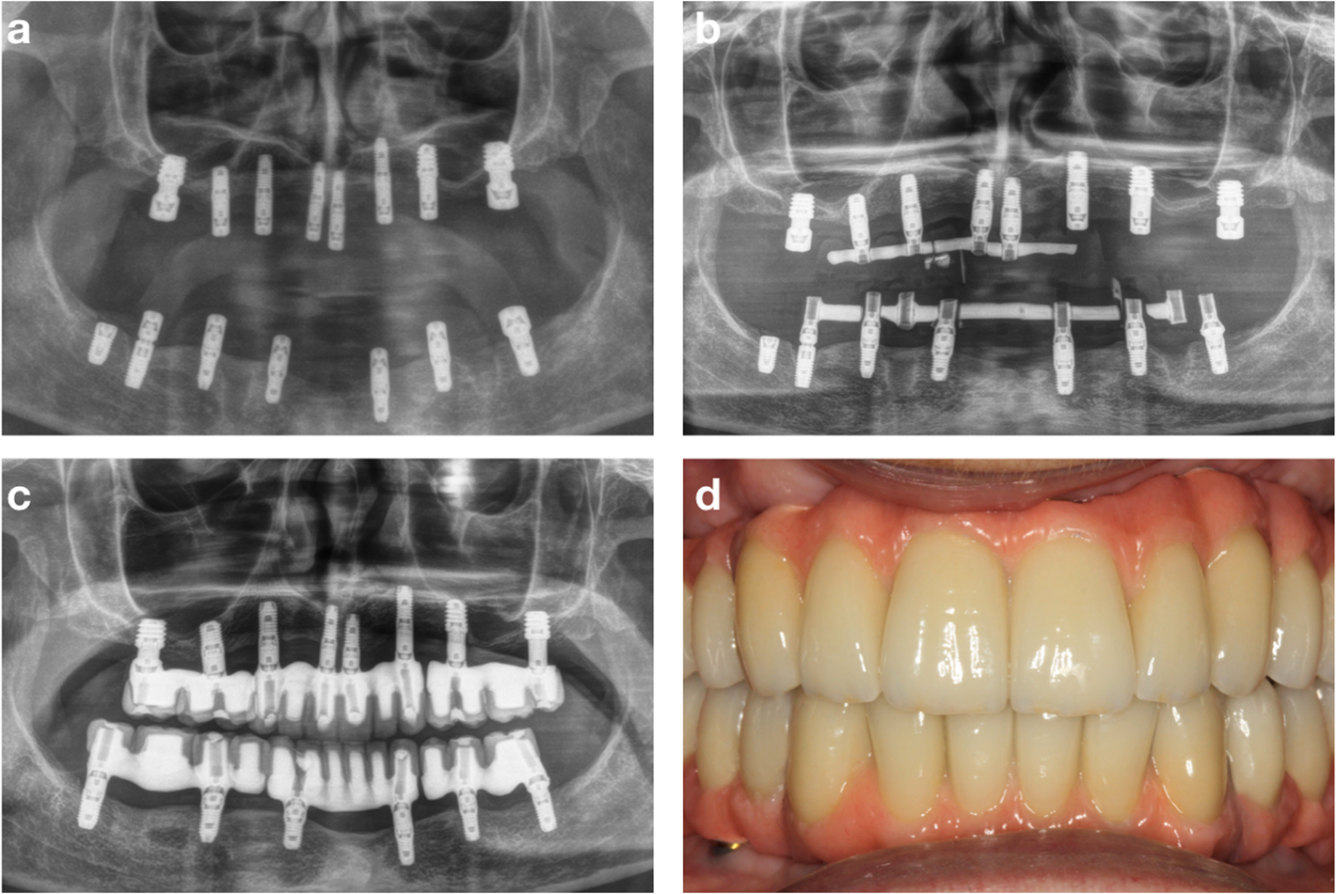
Implant placement surgery after 4 months of healing (**a**). Immediate loading of the new implants and the explanation of the implant at the left first molar (**b**). Panoramic radiograph showing the case finished with 12 months of follow-up (**c**). Clinical image showing the definitive screw-retained prostheses (**d**)

Table 1 shows the relevant available information regarding the fractured dental implants. Ten dental implant fractures were observed in 7 patients. There were no differences regarding the location of the fractured implants, as 40% were located in the maxilla and 60% were located in posterior areas (Table 1). Postoperative recovery of the patients was uneventful and pain was successfully managed by oral analgesics. Figures 3 and 4 show one clinical case that was treated with the counter-torque technique to remove failed dental implants.

**Table 1:** Description of the implants fractured during the explantation

Patient	Location	Manufacturer	Abutment connection	Type of fracture	Location of the fracture	Category of bone support around the implant (%)	Use of trephine bur
1	25	Unknown	Internal	Complete fracture	Implant body (apical third)	76–100	No
	14	Unknown	Internal	Complete fracture	Implant body (apical third)	76–100	Yes
2	15	Nobel	Internal	Fissure line	Implant neck	51–75	Yes
3	14	Nobel	External	Fissure line	Implant neck	26–50	No
	37	Nobel	External	Fissure line	Implant neck	0–25	No
4	46	Nobel	Internal	Fissure line	Implant neck	51–75	Yes
5	34	Modus	External	Fissure line	Implant neck	76–100	No
6	31	Biotech	Internal	Complete fracture	Implant body (apical third)	0–25	No
	32	Biotech	Internal	Complete fracture	Implant body (apical third)	26–50	No
7	43	Unknown	External	Complete fracture	Implant body (apical third)	51–75	No

## Discussion

To our knowledge, this is the study with the largest sample size reporting the performance, in the clinical practice, of the application of counter-torque technique for the removal of nonmobile dental implants. In this retrospective analysis, the counter-torque method demonstrated to be highly predictable, showing a high success rate. The fracture of the implant (0.65% at the implant apical third) was the only detected complication. The principle mechanism of implant explantation was the application of shear stress to break the osseointegration of the implant. Atraumatic implant explantation could be achieved. Besides, this technique demonstrated to be efficient in the removal of nonmobile implants surrounded by different bone levels, ranging from 0–25% to 76–100%.

Due to the low percentage of explantation failures with the counter-torque technique detected in this cohort and the retrospective design of this study, it has not been possible to detect risk factors that could be associated with implant fracture during the explantation or the need to use trephine burs. The influence of the bone quality in the implant removal could not also be assessed. Thus, future prospective studies would be useful to establish a scientifically sound protocol for dental implant explantation using the counter-torque strategy.

Nevertheless, the procedure described in this report has shown to be efficient and safe for the atraumatic removal of nonmobile dental implants and preserving the tissues [6, 7].

Based on previous publications, this strategy enables the direct installation of a dental implant in the extraction site which could maximize the time and cost-efficiency of the treatment [8, 9].

## Conclusions

The good performance of the counter-torque method, as an atraumatic alternative for nonmobile implant removal, reinforces its use in the clinical practice. However, the technique is not exempt from complications (although at a very low rate).

## Data Availability

Data will not be shared but are available upon request.
